# Inferior ST-Elevation Myocardial Infarction (STEMI) Presenting as Left Otalgia: A Case Report

**DOI:** 10.7759/cureus.108451

**Published:** 2026-05-07

**Authors:** Simon B Bactawar, Airada D Bactawar, Carlos Gaibor, Mohammad Abdul-Waheed

**Affiliations:** 1 Internal Medicine, University of Kentucky, Bowling Green, USA; 2 Cardiology, University of Kentucky, Bowling Green, USA

**Keywords:** angina equivalents, inferior stemi, left ear pain, pci, right coronary artery (rca), severe diabetic neuropathy

## Abstract

Diabetic populations are predisposed to atypical, or sometimes silent, responses to myocardial ischemia. This is often the result of chronic neuropathy involving the nociceptive sympathetic pathways of the heart that converge at the T1-T4/5 spinal columns. When these signals are blunted, ischemic pain signals may be delivered via the "Arnold’s nerve," the auricular branch of the vagus that innervates the external auditory canal.

A 70-year-old woman with a history of type 2 diabetes (diagnosed 14 years before presentation), hypertension, hyperlipidemia, and chronic obstructive pulmonary disease presented to the urgent care for a 24-hour history of left ear pain. Following a negative otoscopic exam, she was advised to apply warm compresses and use Tylenol. The subsequent day, she developed intractable nausea, vomiting, and generalized weakness. Emergency medical services were notified and en route identified an acute inferior ST-elevation myocardial infarction (STEMI) via 12-lead electrocardiogram. Emergent left heart catheterization revealed total right coronary artery occlusion, which resolved after deployment of two drug-eluting stents. Her symptoms of left ear pain and nausea resolved postintervention, and she was discharged the following day. This case thus underscores an exceedingly rare but critical anginal equivalent in diabetics, left otalgia.

## Introduction

Acute coronary syndrome (ACS) encompasses three clinical presentations: ST-elevation myocardial infarction (STEMI), non-ST-elevation myocardial infarction (NSTEMI), and unstable angina. The hallmark symptom for ACS involves chest pain or discomfort, which is typically retrosternal with radiation to the left arm and left jaw, due to the afferent convergence of cardiac sympathetics with somatic pathways at the T1-T5 spinal levels [1,2]. 

However, not all ACS present with chest pain and the aforementioned symptomology. The diabetic population, in particular, is predisposed to silent or painless myocardial infarction. This is the result of chronic hyperglycemia-mediated autonomic/sympathetic neuropathy that blunts the primary T1-T5 pathway. These signals seek alternative routes of atypical presentations or anginal equivalents [3]. 

While atypical symptoms for STEMI, such as epigastric discomfort, nausea, emesis, and diaphoresis, are well-documented, isolated left ear otalgia as the primary initial symptom of myocardial ischemia remains exceedingly rare. The likely mechanism involves the neuroanatomy of the vagus nerve, specifically at the nucleus tractus solitarius, through convergence of myocardial ischemia distress signals with external auditory canal afferent somatics [4]. Here, we report the case of a 70-year-old diabetic female whose initial complaint of a right coronary artery (RCA) STEMI was isolated left ear pain. 

## Case presentation

A 70-year-old woman with a 14-year history of uncontrolled type 2 diabetes, with longitudinal HbA1c levels ranging from 7.2%-9.6%, hypertension, hyperlipidemia, and chronic obstructive pulmonary disease, has been experiencing sharp, intermittent left-sided ear pain for approximately three days leading up to her admission. The ear pain was so severe that she went to urgent care (day 2 of ear pain), thinking she might have an acute ear infection. She denied fevers, chills, cough, sore throat, and body aches. Otoscopic examination there, per her report, was unremarkable, and she was sent home with recommendations for symptomatic care with warm compress and acetaminophen therapy. Over the span of 24 hours, she developed intractable nausea and emesis, with weakness of the left arm prompting Emergency Medical Services (EMS) notification. En route to the hospital, her vital signs were stable, with a blood pressure of 148/75, a pulse of 65, a respiratory rate of 18 on room air, and an oxygen saturation of 96%. The in-field 12-lead electrocardiogram (EKG) (Figure 1) raised concern for an acute inferior ST-segment elevation myocardial infarction (STEMI), and the cardiac catheterization lab was activated. Repeat EKG on arrival to the emergency department (Figure 2) confirmed inferior STEMI. Comparison baseline EKG is shown in Figure 3 (one year before admission). Her hematology and renal function panel did not demonstrate anemia, electrolyte disturbances, or renal impairment (Table 1). Her point-of-care glucose was elevated at 183, likely from her uncontrolled diabetes, with a hemoglobin A1c of 7.6 at this admission. Her high sensitivity troponin was >1000 ng/L (reference range 2.9-13 ng/L), with troponin I of 84.9 (reference range 0-0.034 ng/mL). 

**Figure 1:**
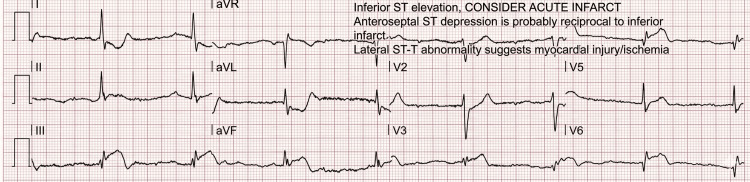
A 12-lead electrocardiogram obtained en route to the hospital demonstrating ST elevations in leads III and aVF with reciprocal changes in I and aVL and anteroseptal leads (V1-V3), consistent with acute inferior ST-elevation myocardial infarction

**Figure 2:**
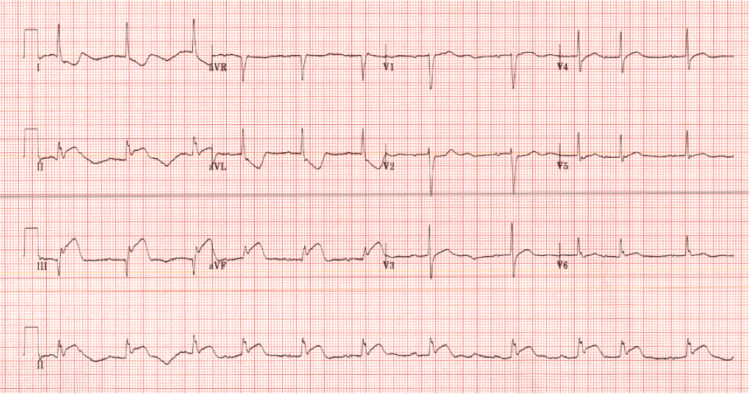
A 12-lead electrocardiogram obtained in the emergency department confirming acute inferior ST-elevation myocardial infarction with ST elevations in leads II, III, and aVF with reciprocal changes in I and aVL

**Figure 3:**
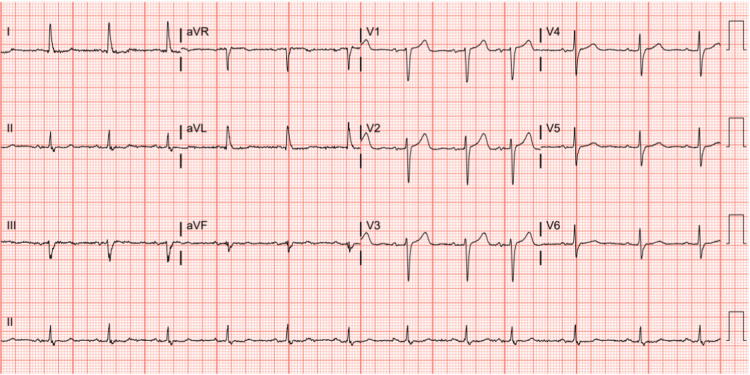
Baseline 12-lead electrocardiogram obtained one year prior demonstrating normal sinus rhythm without ST-segment abnormalities

**Table 1:** Laboratory results at initial presentation to the hospitalization with associated reference ranges Units of measurement used: K/µL: thousands per microliter; M/µL: millions per microliter; g/dL: grams per deciliter; fL: femtoliters; mmol/L: millimoles per liter; mg/dL: milligrams per deciliter

Assay/test	Patient's lab result at presentation	Reference range
White blood cell count	6.9 K/uL	4.8-10.8 K/uL
Red blood cell count	4.65 M/uL	4.2-5.4 M/uL
Hemoglobin	14.5 g/dL	12-16 g/dL
Hematocrit	44.1%	37-47%
Mean corpuscular volume	95 fL	81-99 fL
Platelet count	190 K/uL	140-440 K/uL
Sodium	141 mmol/L	137-145 mmol/L
Potassium	4.4 mmol/L	3.5-5.1 mmol/L
Chloride	105 mmol/L	98-107 mmol/L
Carbon dioxide/bicarbonate	28 mmol/L	22-30 mmol/L
Blood urea nitrogen	15 mg/dL	7-17 mg/dL
Creatinine	0.75 mg/dL	0.52-1.04 mg/dL
Glucose	183 mg/dL	74-106 mg/dL
Magnesium	1.8 mg/dL	1.6-2.3 mg/dL

During cardiac catheterization, the patient was found to have a 100% proximal right coronary artery thrombotic occlusion status post-percutaneous coronary intervention (PCI) with two Xience drug-eluting stents (DES) deployed in an overlapping fashion, 3.5 mm x 23 mm and x 8 mm (Figures 4-5). 

**Figure 4:**
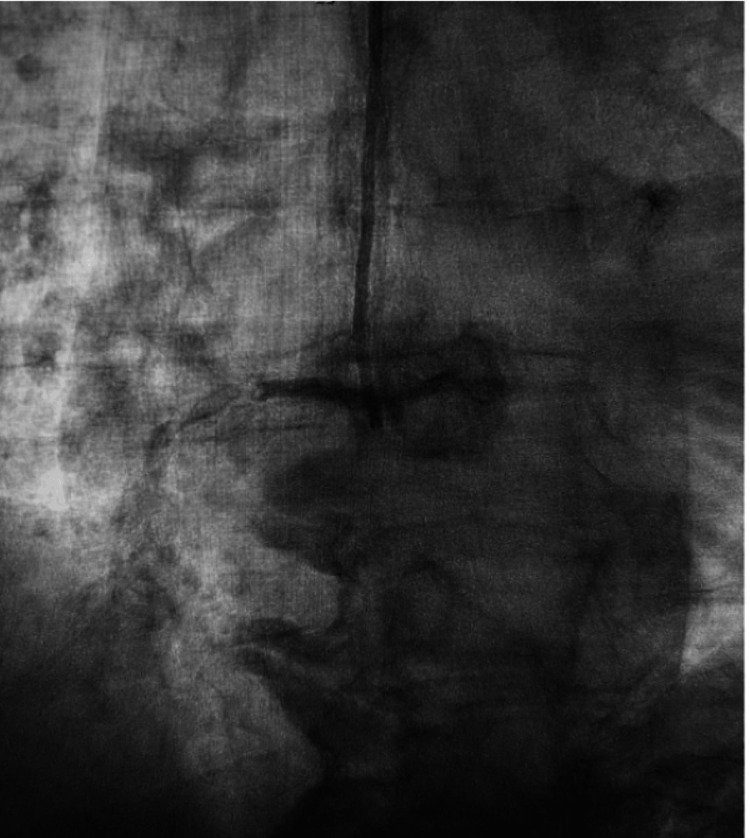
Coronary angiogram demonstrating total occlusion of the RCA, consistent with the electrocardiogram finding of inferior ST-elevation myocardial infarction RCA: right coronary artery

**Figure 5:**
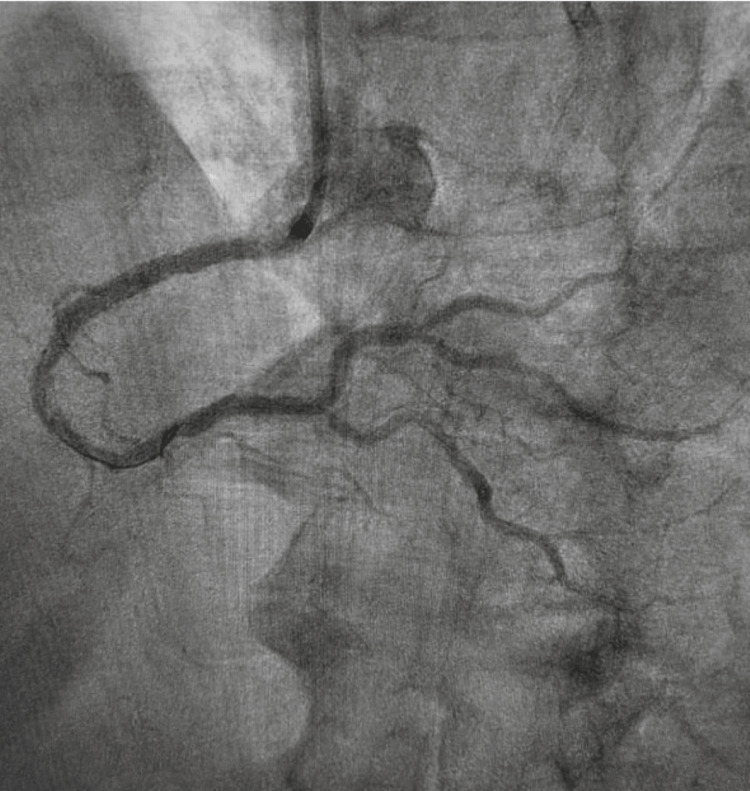
Postintervention (drug eluting stent x2) coronary angiogram demonstrating flow restoration to the RCA and its distal perfusion branches RCA: right coronary artery

Post-PCI EKG (Figure 6) demonstrated improvement of the ST-segment parameter, and a complete transthoracic echocardiogram demonstrated normal left ventricular ejection fraction (55-60%) without valvulopathy.

**Figure 6:**
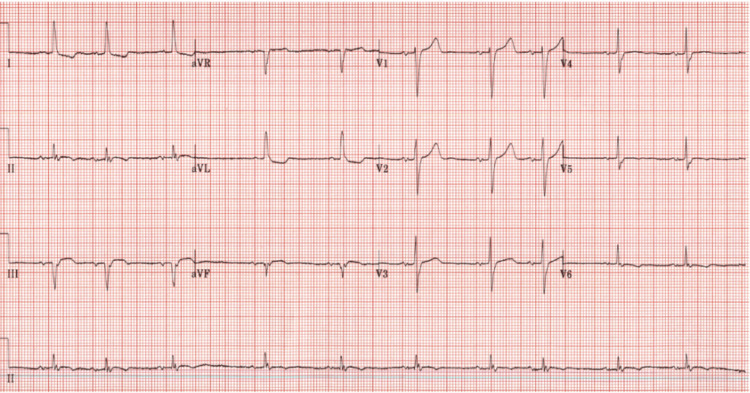
Post-PCI 12-lead electrocardiogram demonstrating resolution of dynamic ST-segment elevation in the inferior leads (II, III, and aVF) PCI: percutaneous coronary intervention

Subjectively, post-PCI, the patient's ear pain and nausea/vomiting resolved, and she was discharged home the subsequent day (a flowchart summarizing the patient's clinical course is outlined in Figure 7). 

**Figure 7:**
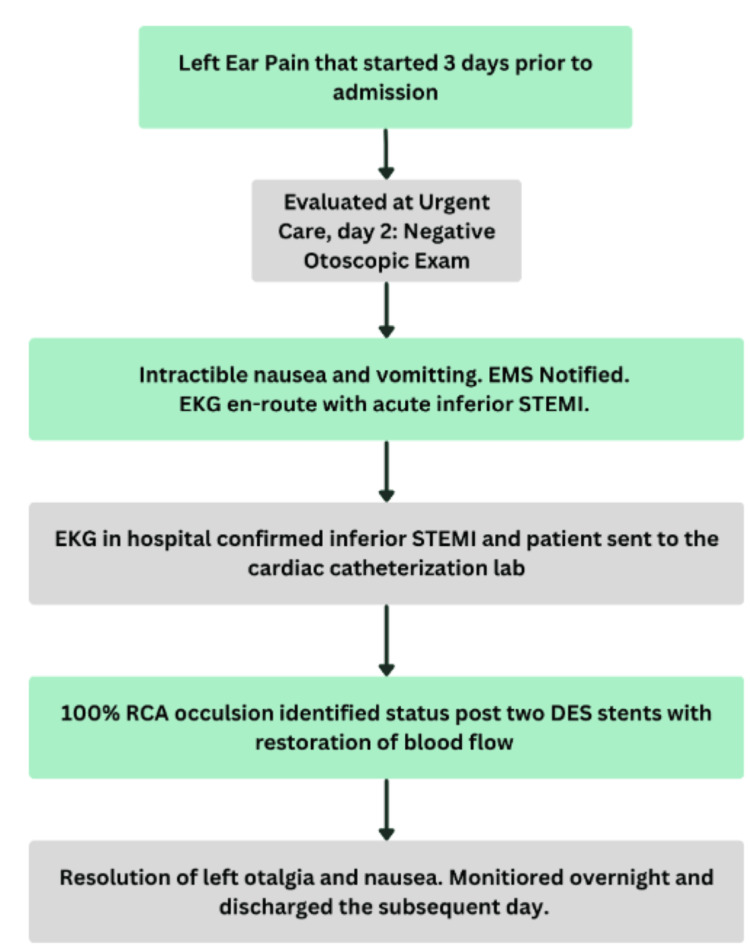
Flowchart showing the chronological summary of the patient's clinical course from the onset of left otalgia to hospital discharge EMS: Emergency Medical Services; EKG: electrocardiogram; STEMI: ST-elevation myocardial infarction; RCA: right coronary artery; DES: drug-eluting stent Image credit: Created by Bactawar SB using Canva (Canva Inc., Perth, Australia)

## Discussion

Isolated left ear otalgia as a manifestation of myocardial ischemia remains a rare but critical anginal symptom. This presentation poses a challenge for acute MI diagnosis as it skews away from the classical retrosternal chest discomfort and traditional left arm/jaw radiation. The explanation of this phenomenon involves the complex neuroanatomical network of the vagus nerve. 

The heart nociceptive afferents travel via the spinal cord sympathetic pathway at levels of T1 to T5 [2]. The spinal sympathetics, via a convergence-projection mechanism, result in the classic chest and arm radiation/involvement in acute myocardial distress [5]. Patients with diabetes, however, particularly in suboptimally controlled and long-standing cases, demonstrate a blunted chest pain and sometimes a silent response to myocardial ischemia [3,6]. This is the result of chronic progressive neuropathy of the sympathetic sensory pathway [3,6]. In these instances, the signal follows a previously subclinical referred pathway involving the vagus nerve/cranial nerve X to the dorsal medial part of the nucleus tractus solitarus, particularly responsible for cardiac reflexes and pain response [1,4]. The vagus nerve also supplies the sensory pathway for the external auditory canal via the auricular branch, classically referred to as ‘Arnold’s Nerve’ (after Friedrich Arnold, a German anatomist) [1].

The pathophysiology of referred otalgia from myocardial infarction can be explained by the convergence of cardiac pain afferents and auricular somato-sensory afferents within the nucleus tractus solitarus (NTS) [4,7,8]. This convergence link has been highlighted in a few published case reports: for example, Ugalde et al., in their report, documented a 47-year-old man with bilateral ear pain whose presenting EKG demonstrated nonspecific ST changes and Q waves from a prior myocardial infarct; subsequent troponin levels were critically elevated, prompting left heart catheterization, which revealed angiographically significant blockages in the obtuse marginal second and fourth branches. After PCI, the patient's otalgia resolved [1]. 

Furthermore, inferior wall MI (involving the RCA) is classically associated with vagal tone dominance, resulting in nausea, emesis, and transient sinus bradycardia [8,9]. The accompanying symptoms that our patient exhibited, as well as the atypical left ear otalgia, were of vagal etiology, further supporting the vagal-mediated convergence mechanism. 

As observed in the aforementioned case report by Ugalde et al., the prompt resolution of both left ear otalgia and nausea/emesis status post stenting of the culprit right coronary artery further identifies this presentation as a rare anginal equivalent. This case thus demonstrates that clinical suspicion for acute coronary syndrome should be maintained when patients with longstanding and uncontrolled diabetes present with unexplained left ear otalgia and an unremarkable otoscopic examination. Such presentations should prompt further investigation via an electrocardiogram and cardiac biomarker/troponin assay to avoid diagnostic delays. 

## Conclusions

This case highlights isolated left otalgia as a rare but critical anginal equivalent presentation of inferior STEMI n in a patient with longstanding, uncontrolled type 2 diabetes. It also highlights the vagal-mediated referred/alternative pathway that myocardial nociception can be redirected during ischemic distress due to chronic autonomic neuropathy, blunting of the classic/primary T1-T5 cardiac sensory pathway. For this, clinicians should maintain a high index of suspicion for acute coronary syndromes in diabetic patients presenting with otoscopically unremarkable otalgia, particularly with concomitant vagal symptoms such as nausea and emesis, to avoid critical diagnostic and revascularization delays. 

## References

[1] Ugalde I, Anjum I, Lo Presti S, Tolentino A (2018). Myocardial infarction presenting as ear fullness and pain. J Investig Med High Impact Case Rep.

[2] Kapa S, DeSimone CV, Asirvatham SJ (2016). Innervation of the heart: an invisible grid within a black box. Trends Cardiovasc Med.

[3] Draman MS, Thabit H, Kiernan TJ, O'Neill J, Sreenan S, McDermott JH (2013). A silent myocardial infarction in the diabetes outpatient clinic: case report and review of the literature. Endocrinol Diabetes Metab Case Rep.

[4] van Weperen VY, Vaseghi M (2023). Cardiac vagal afferent neurotransmission in health and disease: review and knowledge gaps. Front Neurosci.

[5] DeVon HA, Ryan CJ, Ochs AL, Shapiro M (2008). Symptoms across the continuum of acute coronary syndromes: differences between women and men. Am J Crit Care.

[6] Marchant B, Umachandran V, Stevenson R, Kopelman PG, Timmis AD (1993). Silent myocardial ischemia: role of subclinical neuropathy in patients with and without diabetes. J Am Coll Cardiol.

[7] Dundar R, Kulduk E, Kemal Soy F, Sengul E, Ertas F (2014). Myocardial infarction as a rare cause of otalgia. Case Rep Otolaryngol.

[8] Schismenos V, Tzanis AA, Papadopoulos GE, Nikas D, Koniari I, Kolettis TM (2023). Autonomic responses during acute anterior versus inferior myocardial infarction: a systematic review and meta-analysis. Cureus.

[9] Kawasaki T, Akakabe Y, Yamano M (2009). Vagal enhancement as evidence of residual ischemia after inferior myocardial infarction. Pacing Clin Electrophysiol.

